# Linking Structure and Optical Properties of Plasmonic Nanoparticles on Tunable Spherical Surfaces

**DOI:** 10.1021/acsami.5c11151

**Published:** 2025-08-11

**Authors:** Francesco Brasili, Angela Capocefalo, Giovanni Del Monte, Rodrigo Rivas-Barbosa, Javier Pérez, Edouard Chauveau, Federico Bordi, Carlo Rizza, Domenico Truzzolillo, Emanuela Zaccarelli, Simona Sennato

**Affiliations:** † 96975 Institute for Complex Systems, National Research Council, Piazzale Aldo Moro 5, Rome 00185, Italy; ‡ Department of Physics, 9311 Sapienza University of Rome, Piazzale Aldo Moro 5, Rome 00185, Italy; § Department of Physical and Chemical Sciences, 201850 University of L’Aquila, Via Vetoio, Coppito, L’Aquila 67100, Italy; ∥ Soft Condensed Matter and Biophysics, Debye Institute for Nanomaterials Science, Utrecht University, Princetonplein 1, Utrecht, CC 3584, The Netherlands; ⊥ Synchrotron SOLEIL, L’Orme des Merisiers Départementale 128, Saint-Aubin 91190, France; ▶ Laboratoire Charles Coulomb, UMR 5221, 131799 CNRS−Université de Montpellier, Montpellier 34095, France

**Keywords:** soft colloids, thermoresponsive microgels, nanoparticles, plasmon coupling, plasmon ruler, two-dimensional curved interface, stimuli-responsive optical materials

## Abstract

The complexation of plasmonic nanoparticles (NPs) and thermoresponsive microgels is widely recognized as a powerful route to realize hybrid systems with tunable optical properties for different applications. At the same time, it provides a unique experimental platform to investigate the physics of NP organization on curved two-dimensional surfaces, a fundamental problem with implications spanning from biology to materials science yet unexplored at the nanoscale. However, a microscopic description of the mechanisms governing the spatial organization of the NPs and their rearrangement across the microgel volume phase transition (VPT) is lacking so far. Combining small-angle X-ray scattering and state-of-the-art simulations, we uncover how the microgel VPT controls NP-NP interactions, showing that temperature-induced microgel collapse drives a redistribution of NPs toward the periphery, with a tendency to order on the spherical surface. Moreover, we quantitatively reproduce both the structural and optical experimental data through a simple toy model, ultimately establishing for the first time a direct link between the interparticle distance and plasmon coupling. Our study paves the way for experimentally investigating phase transitions on tunable curved surfaces at the nanoscale, achieving fine control of their plasmonic response.

## Graphical abstract

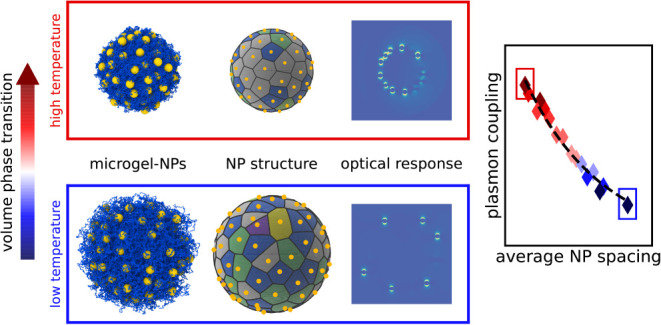

## 1 Introduction

The study of particle systems confined to curved two-dimensional interfaces is a fundamental question in physics, dating back to the classical Thomson problem.^1^ Its straightforward generalization to various natural and synthetic systems, including viral capsids, biological membranes, two-dimensional crystals, colloidosomes, and advanced nanomaterials for photonic and electronic applications, further broadens its relevance.^2–5^ While extensive theoretical studies have explored the equilibrium configuration and thermodynamics of particles on curved surfaces,^6–10^ experimental investigations of particle organization under two-dimensional curved confinement have been limited to the microscale^11–14^ while being still completely absent at the nanoscale. Addressing this gap is essential as interparticle interactions at the nanoscale deviate from classical additivity,^15^ potentially giving rise to unexpected behaviors distinct from those observed at the microscale and predicted by current numerical models. Furthermore, curved surfaces have often been treated as passive geometric constraints that merely dictate particle arrangement. In reality, biological interfaces can actively interact with adsorbed particles via specific binding mechanisms or feedback processes, including curvature modification, as observed in cellular deformation.^16^ To tackle these challenges, we investigated an experimental model system where the spherical constraint is provided by thermoresponsive microgels. These soft polymeric colloids act as a dynamic substrate onto which nanoparticles (NPs) are electrostatically adsorbed. Specifically, we leverage their volume phase transition (VPT), namely a reversible collapse triggered by temperature increase^17^ to tune the curvature of their external surface, triggering the rearrangement of the adsorbed NPs. The subwavelength characteristic dimensions of the microgel–NPs complexes prevent direct imaging via optical microscopy, which has hitherto been the standard method for studying colloidal particles at the curved interface of Pickering emulsions.^18^ To overcome this limitation, we combine small-angle X-ray scattering (SAXS) experiments with molecular dynamics simulations, enabling us to rationalize the temperature-dependent arrangement of adsorbed NPs. As a proof of concept, we focus on a plasmonic system using gold NPs as interacting colloids to have an additional local probe to monitor interparticle interactions via the localized surface plasmon resonance (LSPR) and its modification induced by plasmon coupling. In this respect, a pioneering contribution was put forward by Gawlitza et al.,^19^ who recognized the effect of the microgel structure on NP loading and on plasmon coupling, later extensively exploited to engineer responsive photonic nanomaterials.^20–24^ However, a microscopic understanding of NP rearrangement and a quantitative connection between their structure and the resulting optical properties have not been established so far.

In this work, we fill this gap by investigating the temperature-dependent arrangement of adsorbed gold NPs and linking, for the first time, their structural organization to their optical response via extinction spectroscopy and electromagnetic full-wave simulations. We find that as temperature increases, the NPs approach each other due to microgel shrinkage but do not form clusters or dimers. Instead, they rearrange to minimize electrostatic repulsion by maximizing their geodesic distance. We also detect, at high temperatures, an increased tendency toward NP ordering on the microgel spherical surface. To interpret the optical properties in light of these findings, we introduce a toy model describing NPs confined to a spherical shell that accurately captures the experimental structure factors at all temperatures. We then incorporate the so-calculated NP arrangement into electromagnetic simulations and quantitatively reproduce their extinction spectra. We finally establish a direct relationship between plasmon coupling strength and NP separation, independent of the total number of employed NPs, laying the groundwork for precise control over the optical properties of soft plasmonic complexes.

## 2 Results and Discussion

### 2.1 Optical and Structural Properties of the Microgel-NPs Complexes across VPT

We employ cationic poly­(*N*-isopropylacrylamide) (pNIPAM) microgels interacting with anionic, spherical gold NPs and analyze the samples by extinction spectroscopy and SAXS for different temperatures *T* across the VPT. Extinction spectra, reported in Figure 1A, for the NP/microgel number ratio *n* = 150, show that the LSPR evolves with increasing *T*, exhibiting a redshift, slight quenching, and an increase in the extinction at larger wavelengths. Similar results are found for *n* = 300 (Figure S1). These spectral changes are the signature of plasmon coupling, which is the activation of low-energy collective modes arising from plasmon hybridization^25^ occurring when, driven by the microgel deswelling, NPs approach each other, reaching surface-to-surface distances *d* of a few nanometers. Note that NPs alone do not aggregate with *T*, as shown in Figure S1. Even if the shift of the LSPR alone gives a good description of the optical changes across the VPT and can be used to connect the optical properties of microgel–NPs complexes and the structure of the polymer network,^26^ it does not reveal any information about the broadening of the peak and the onset of coupled plasmon modes at higher wavelengths, which are the main features of interest for many applications, such as colorimetric detection or surface-enhanced spectroscopies.^27–31^ To account for these spectral modifications, we therefore define the degree of coupling as the spectral weight Δ­(*A*_*C*_/*A*_*tot*_) of the region between 570 and 800 nm (eq 1 and the inset of Figure 1B). Notably, as shown in Figure 1B, Δ­(*A*_*C*_/*A*_*tot*_) follows a sigmoidal trend in temperature, with an inflection point occurring at *T*_*C*_ ≃ 33.7^◦^C, consistent with the value obtained from the swelling curves of the hydrodynamic radius (Figure S2), demonstrating the possibility of detecting the VPT of the microgels through the coupling of the adsorbed NPs.

**Figure 1:**
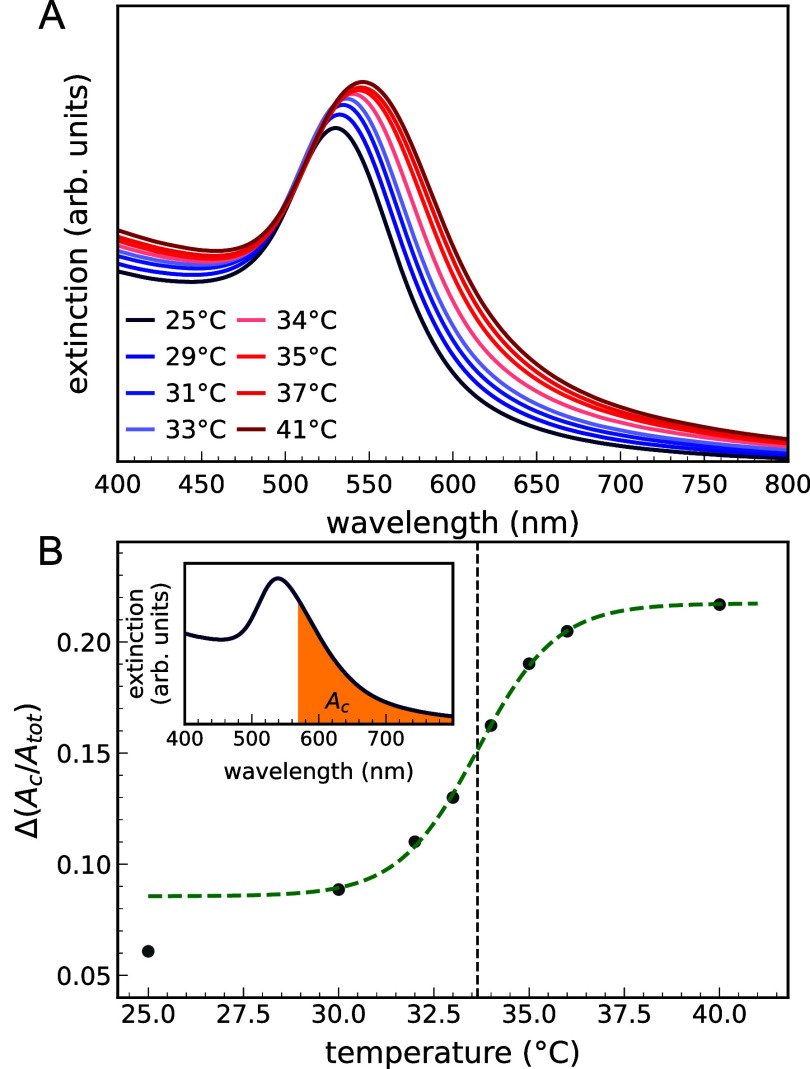
Optical properties of the microgel–NPs complexes: (A) extinction spectra for *n* = 150 and different temperatures *T*, and (B) degree of coupling Δ­(*A*_*C*_/*A*_*tot*_) as a function of *T*; the sigmoidal fit (dashed line) yields a critical temperature *T*_*C*_ = 33.7 ± 0.2^◦^C. The region of coupled plasmon modes used to define Δ­(*A*_*C*_/*A*_*tot*_) (eq 1) is shown in the inset (orange, A_c).

To gain knowledge of the microscopic organization of the NPs, we acquired electron microscopy images of the samples and measured NP–NP structure factors *S*(*q*) as a function of *T*, as reported in Figure 2 for *n* = 150. Samples for imaging were prepared at two controlled temperatures, below and above the microgel VPT, to visualize possible morphological changes. The NPs show a clear tendency to localize in the peripheral regions of the corona, a behavior that becomes more pronounced at higher temperatures. Moreover, by examining the NP distribution across different microgels, we observed low variability throughout the sample (Figure S3A). Similar results were obtained for *n* = 300 (Figure S3B). The measured SAXS intensities solely arise from NPs due to the much higher contrast of gold with respect to the polymer.^20^ Therefore *S*(*q*)’s are directly obtained by dividing each scattering curve by the form factor of NPs (Figure S4). The complex structure of the soft assemblies makes the interpretation of the curves and their *T*-dependent evolution far from trivial within the analyzed *q*-range. In particular, the onset of a new peak at ∼1 × 10^–2^ Å^–1^ (highlighted in light blue) when the temperature exceeds *T*_*C*_ = 33.3 ± 0.3°C (swelling curve in Figure S2) and the concurrent shift of the large band from 1.2 × 10^–2^ Å^–1^ to 2.2 × 10^–2^ Å^–1^ point to a nanoscale rearrangement of NPs across the VPT of the microgels.

**Figure 2:**
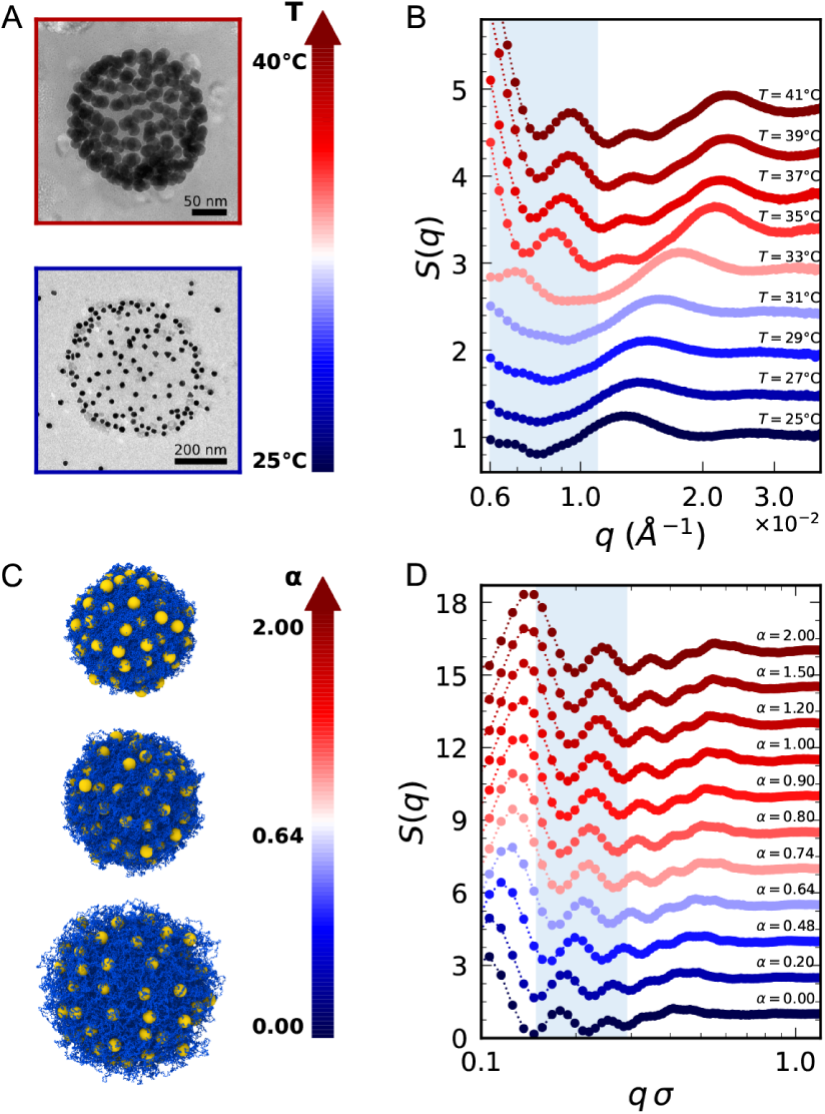
Structural characterization of microgel–NPs complexes. (A) Electron microscopy images of two representative complexes deposited at controlled temperatures below and above the VPT. (B) Experimental structure factors *S*(*q*) of NPs adsorbed to microgels at different *T*. (C) Simulation snapshots at the same magnification for three representative values of the effective temperature α; blue and yellow particles represent microgel monomers and NPs, respectively. (D) Numerical *S*(*q*) of NPs adsorbed to a microgel with *N* = 112*k* and *f* = 0.02 for *n* = 150 and different α values. Curves in panels (B) and (D) are vertically shifted for clarity; the light blue color highlights the peak mostly described in the text.

To decipher these observations, we resort to molecular dynamics simulations of a well-established microgel model^32^ that provides a realistic description across the VPT^33^ also in the presence of charged monomers^34^ and upon interactions with charged NPs^35^ (see Methods for details). In the simulations, the increase in temperature is reproduced by the use of a solvophobic potential between the monomers (eq 2), which implicitly accounts for monomer–solvent interaction through a parameter α that plays the role of an effective temperature.^36^ Good solvent conditions correspond to α = 0, while the affinity to the solvent gets worse as α increases. Simulations are conducted for single microgels, made of either *N* = 14*k* or *N* = 112*k* monomers of diameter σ, with a varying fraction *f* of charged monomers located on the external shell^34^ for different values of α and in the presence of varying amounts of NPs. The swelling curve of the microgel (Figure S5A) displays the occurrence of the VPT at α_*C*_ ∼0.64. The snapshots of the microgel–NPs complexes (Figure 2C) show that, also in the simulations, NPs tend to adsorb to the microgel corona, attracted by charged monomers,^35^ and localize more and more externally as α increases. We analyze the simulations for *N* = 112*k* and *n* = 150 to calculate NP–NP structure factors and show in Figure 2D the *q*-range where they exhibit very similar features to experiments: high oscillations at low *q* followed by smaller peaks at intermediate *q* and a general shift of the peaks toward higher *q* with increasing temperature. Full *q*-range *S*(*q*) values are reported in Figure S5B, comparing the extreme values α = 0 and α = 2. At low *q* (below ∼ 0.3σ^–1^), where the features of the overall microgel–NPs system emerge, the curves are well captured by the form factor of a spherical shell, supporting the hypothesis that NPs are distributed only within the microgel corona. At higher *q*, the peak around 0.2σ^–1^ (highlighted in light blue in Figure 2D) is already present below the VPT, in contrast to the corresponding one in experiments, at ∼ 1 × 10^–2^ Å^–1^. This result is found in all performed simulations, either varying *f* and *n* or modifying the microgel size (Figure S6). Moreover, we did not detect evident NP structures, such as clusters or regular arrays, in the snapshots. Given these observations, we conclude that the modifications of both the experimental and numerical structure factors may be the hallmark of subtle structural modifications at the scale of NP–NP interactions, which deserves in-depth investigation in order to establish a connection with the modulation of plasmon coupling.

### 2.2 Numerical Insights on NP Organization on the Spherical Surface

To shed light on the different features of the structure factor in the intermediate and high *q*-range and their variations across the VPT, we first analyze the shift of all peaks toward larger *q* values by filtering out the effect of microgel deswelling due to the VPT. We thus plot *S*(*q*) as a function of *qR*_*H*_(*T*) in Figure 3A,B, for experiments and simulations, respectively, with *R*_*H*_(*T*) being the hydrodynamic radius at each temperature. In this way, it becomes evident that the region *qR*_*H*_ ≤ 11, where the curves above the VPT have a peak, falls outside the experimental *q*-range below the VPT. Therefore, the peak should already be present at low temperatures, similar to what is found in simulations, but its apparent onset at high *T* is simply due to the fixed experimental *q*-window.

**Figure 3:**
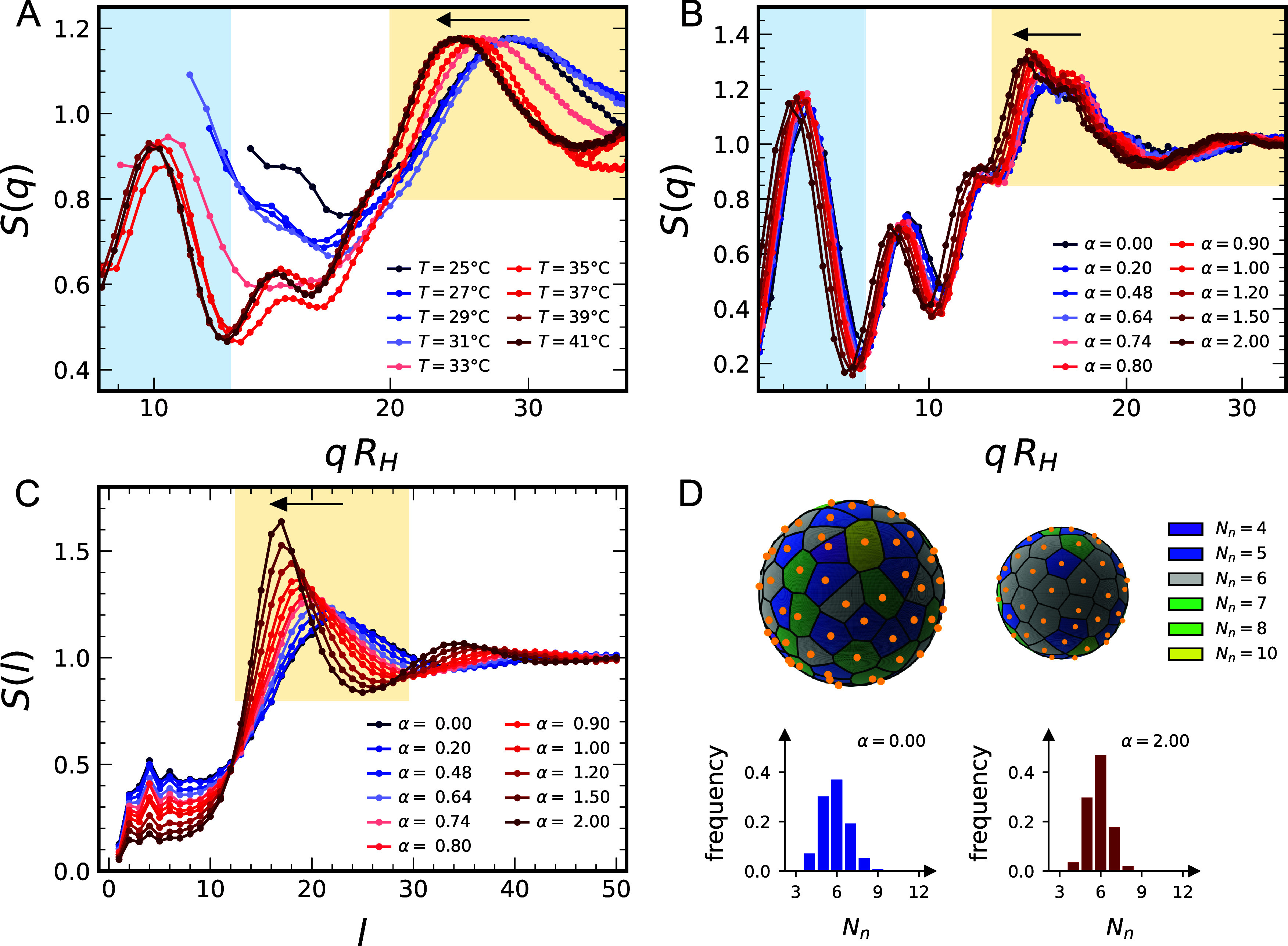
Structural analysis of NPs adsorbed to microgels across the VPT: *S*(*q*) as a function of *qR*_*H*_ for SAXS experiments (A) and numerical simulations (B). The light blue regions are the same as in Figure 2; in panel A, curves are normalized to the maximum. (C) Numerical spherical structure factors *S*( l ) of NPs. The shifts toward low *q* or l values are highlighted by a yellow region. (D) Voronoi tessellation (top) and corresponding distributions of the number *N*_*n*_ of nearest neighbors (bottom), for α = 0 (left) and α = 2 (right). The tessellation is performed on the configurations of adsorbed NPs, projected on the unit sphere. *S*( l ) and distributions of *N*_*n*_ are mediated over 5 different topologies of a microgel with *N* = 14*k* and *f* = 0.161.

Having established this, we take a closer look at Figure 3 where, for both experimental and simulation data, the rescaled curves do not perfectly overlap at different temperatures. Indeed, a shift toward lower *q* values is observed in both panels, pointing to a variation in NP-NP arrangement as a function of *T*. This effect needs to be appropriately interpreted within the spherical geometry constraint imposed on the NPs by the presence of the microgel. Hence, in order to decouple the contribution of the confining geometry to *S*(*q*), we calculate the spherical structure factor *S*( l ), which is defined on the surface of a unit sphere according to eq 4. Here, l is the degree of the spherical harmonics used to represent the particle density on the spherical surface.^6^ It plays a role equivalent to the wave vector *q* in flat space, with the difference that l can only take discrete values since the spherical surface that defines the geometry has a finite size. The resulting *S* ( l ) is reported for different temperatures in Figure 3C (see also Figure S7). The behavior of *S*( l ) is very similar to that of a standard structure factor in bulk, without all the low-*q* oscillations due to the underlying spherical geometry. We thus find that *S*( l ) is characterized by a main peak for l *∼* 18, followed by smooth oscillations. The *T*-dependence of *S*( l ) shows that such main peak becomes sharper, shifting to lower and lower l as *T* increases. Since this calculation is performed on a unit sphere, it is analogous to the rescaled *S*(*q*) reported in Figure 3B, thus confirming the shift observed in experiments and in simulations for the full structure factors. To sum up, the shift to low wavevectors of all structure factors clearly indicates that the NPs increase their relative geodesic distance (on the unit sphere) as the microgels undergo the VPT. This is due to their mutual electrostatic repulsion that becomes stronger and stronger as they get closer due to the underlying shrinking of the microgel. We further assessed the role of electrostatic interactions in determining the NP arrangement and its modifications by simulations varying the NPs’ charge (Figure S7). Furthermore, we note that the present data do not show evidence of a crystalline arrangement of NPs, although the main peak in *S*( l ) visibly sharpens, pointing to an increased ordering on the spherical surface. To further substantiate this observation, we analyzed in more detail the NP configurations by performing a Voronoi tessellation of their positions projected onto the unit sphere. This is a standard method, commonly employed to characterize phase transitions of colloidal particles in complex geometries, particularly on curved surfaces, in terms of the number and arrangement of nearest neighbors.^13,14^ The resulting tessellations, below (α = 0) and above (α = 2) the VPT, are shown in Figure 3D, revealing a clear homogenization in the number of edges per cell, corresponding to the number *N*_*n*_ of nearest neighbors of the associated particle. This observation is supported by the histograms of Figure 3D, which show a marked increase in the frequency of *N*_*n*_ = 6, along with a narrowing of the distribution around this value. On the contrary, the tessellation cells are clearly not regular hexagons, thus excluding the occurrence of full crystallization, consistent with what is observed through *S*( l ). Nevertheless, we set the bases for investigating at the nanoscale the collective phenomena and particle dynamics associated with phase transitions on curved surfaces, which can be accessed by fine-tuning the characteristics of the microgels and NPs.

### 2.3 Connecting the 3D NP Structure to the Plasmonic Properties

To connect the microscopic NP arrangement to the degree of plasmon coupling, we use a numerical toy model, sketched in Figure 4A, inspired by the numerical simulations put forward by Oberdisse and collaborators to study the structure factors of micelles adsorbed to colloidal silica.^38–40^ As detailed in the Methods, the model consists of randomly arranging a set of points, mimicking NPs, within a spherical shell, which represents the external corona of the microgels. We choose this geometrical constraint based on electron microscopy results, on the insights provided by numerical structure factors as well as on previous studies, showing that when microgel charges are introduced only by initiator molecules, as in the present case, they prevalently position themselves in the external corona of the microgel^41^ and confine adsorbed NPs in the same region due to electrostatic attraction.^35^ By using this model, we can vary the features of the system arbitrarily, matching the experimental NP/microgel size ratio, which is not possible within the present simulations. In the toy model, we thus vary four different parameters independently: the external radius *R* and the thickness *t* of the shell, the number of NPs in the shell *N*_*p*_ and the minimum distance *D* + *d*_*min*_ between them, where *D* is the NP diameter and *d*_*min*_ the minimum surface-to-surface distance. We then calculate *S*(*q*) of the NPs within the shell and find that the model clearly allows us to interpret the different features of the measured *S*(*q*) and to discern the influence of each parameter on its behavior (Figures S8 and S9). Specifically, we confirm that the low-*q* features arise from the confinement of particles within a spherical shell, and indeed, they change when *R* and *t* change. Instead, the broader band between 1 and 3 × 10^–2^ Å^–1^, whose maximum at *q*_*p*_ (highlighted by arrows) increases in intensity and shifts to higher *q* values with temperature, does not depend on the spherical geometry but rather on *d*_*min*_ and *N*_*p*_. This band corresponds to the part of the experimental structure factor that more strongly depends on the NPs’ mutual interactions. Figure 4A shows that with an appropriate choice of parameters and by accounting for the experimental polydispersity σ_*R*_, the model well captures the experimental *S*(*q*), particularly matching the positions of the various peaks, even though it assumes a random NP distribution and neglects interaction-induced structural ordering. Despite minor deviations in the intensities of the maxima and minima at low *q*, we conclude from this analysis that the more pronounced peaks occurring at intermediate *q* in experiments are due to the uniforming of the core–corona structure of the microgels as they undergo VPT. Notably, the model yields meaningful values of the parameters for the description of the experimental results and their trend with temperature. Indeed, we find that the external radius of the shell *R* shrinks from 184 to 82 nm, in good agreement with *R*_*H*_ measurements reported in Figure S2, while the shell thickness *t* reduces from 47 to 2 nm. This pronounced shrinking of the shell, much greater than the overall microgel deswelling, is due to the incorporation of NPs,^35^ indicating that, across the VPT, the corona of the microgel compacts much more than the core, thus pushing the NPs outward. The further action of mutual electrostatics finally results in the rearrangement of NPs at overall larger relative distances, also favoring an increase ordering, as revealed by *S*( l ). As further validation of the toy model, we employ the obtained NP configurations to compute the extinction spectra through electromagnetic full-wave simulations, appropriately taking into account the heterogeneous internal microgel structure, as detailed in Figure S10. The resulting maps of electric field enhancement at the wavelength λ = 630 nm are shown in Figure 4B, allowing us to clearly visualize the variations across the VPT. At *T* = 25^◦^C, the NPs on the microgel behave as isolated particles, whereas at *T* = 41^◦^C, the electric field is delocalized over adjacent NPs, as evidenced by the zoom of panel (iv), highlighting the activation of plasmon coupling. The corresponding extinction spectra, shown in Figure 4C, are in excellent agreement with experimental data at both analyzed temperatures, confirming that the proposed model accurately describes both the structural and optical properties of the system.

**Figure 4:**
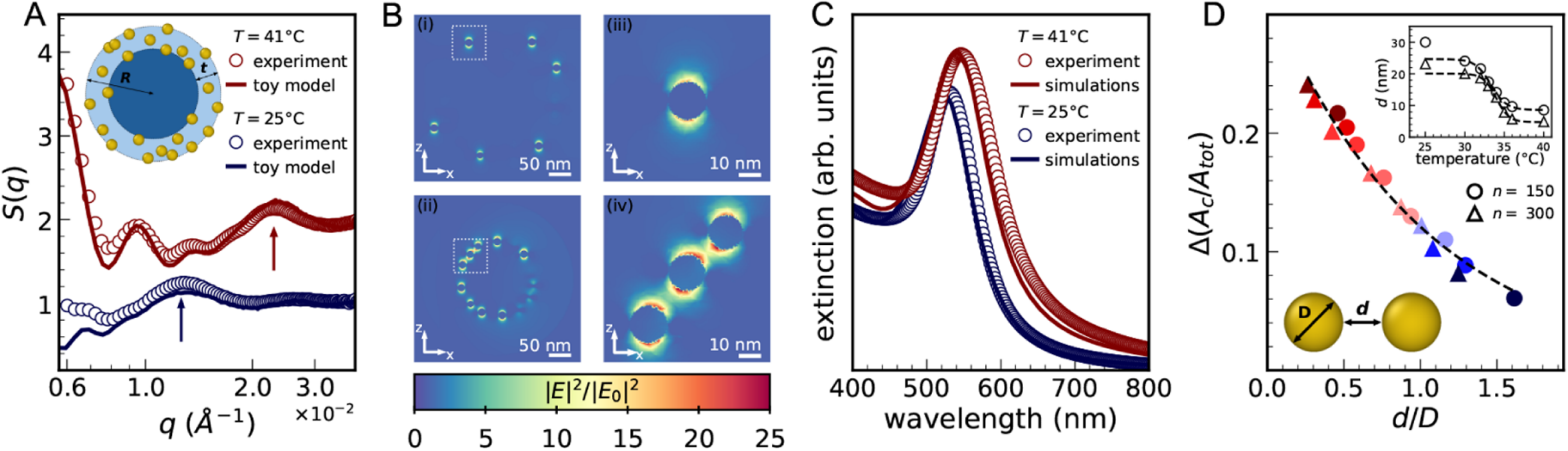
(A) Experimental *S*(*q*) compared to the predictions of the sketched toy model, using *N*_*p*_ = 75, *R* = 184 nm, *t* = 47 nm, *d*_*min*_ = 28 nm, and σ_*R*_ = 6 nm for *T* = 25^◦^C, and *N*_*p*_ = 65, *R* = 82 nm, *t* = 2 nm, *d*_*min*_ = 8 nm, and σ_*R*_ = 6 nm for *T* = 41^◦^C; the arrows identify the position of the peaks *q*_*p*_ corresponding to the distance between NPs. (B) Intensity maps of the electric field enhancement |*E*|^2^/|*E*_0_|^2^, obtained by full-wave numerical simulations of the microgel–NPs complexes, for λ = 630 nm. Cross sections at the planes *y* = 39 nm for *T* = 25^◦^C (i) and *y* = 21 nm for *T* = 41^◦^C (ii). Zoom on a single NP at *T* = 25^◦^C (iii) and on a group of NPs at *T* = 41^◦^C (iv), highlighting the localization of plasmon coupling. (C) Simulated extinction spectra at *T* = 25^◦^C and *T* = 41^◦^C (dashed lines) compared with experimental ones. (D) Degree of coupling Δ­(*A*_*c*_/*A*_*tot*_) as a function of *d*/*D*, with *d* the average surface-to-surface distance and *D* the NP diameter, for *n* = 150 and *n* = 300 (same color-coding as in Figure 3A). The dashed line is a fit to an exponential decay.^37^ Inset: *d* as a function of *T*.

Based on these results, we can finally connect the experimental *S*(*q*) with the optical properties of the complexes as the microgels undergo the VPT. Starting from the position *q*_*p*_ of the first peak of the effective structure factor of the NPs, which is not related to the spherical confinement but reflects the local interparticle arrangement (see Figure 4A), we determine the average surface-to-surface distance between nearest neighbors *d* = 2π/*q*_*p*_ – *D*, with *D* = 18.6 nm being the NP diameter (Figure S4) reported as a function of *T* in the inset of Figure 4D. We then report the degree of coupling Δ­(*A*_*C*_/*A*_*tot*_), obtained from the extinction spectrum at the same *T*, as a function of the ratio *d*/*D* to quantify the plasmon interaction strength^37^ (Figure 4D). Since NPs approach each other more and more upon shrinking of the microgels above the VPT, they are able to explore distances of just a few nanometers between their surfaces and thus give rise to plasmon coupling. Notably, we find that the data for the two studied values of *n* follow the same trend, compatible with an exponential decay pattern^37^ with a characteristic length ∼20 nm. Altogether, these results shed light for the first time on the direct relationship between the coupling of plasmonic NPs adsorbed to thermoresponsive microgels and their average interparticle distance. This enables the optical measurement of the NP-NP nanoscale distances on the spherical surface as a function of temperature, thus serving as a plasmonic “nano-ruler,” similar to those based on DNA technology.^42^ Our study thus opens the possibility to rationally design thermoresponsive plasmonic systems with optical properties tailored to specific needs. For instance, by adjusting the chemical composition of microgels and NP adsorption, this system can serve as a reactor with thermally tunable catalytic activity^43,44^ with the additional advantage of real-time monitoring of NP distances. Beyond these practical implications, our study provides a paradigm shift in the use of thermoresponsive microgels, which we envision as a tunable substrate for colloidal assembly on a curved two-dimensional surface, opening the way to experimentally tackle this important physical problem at the nanoscale.

## 3 Conclusions

The present work reports a detailed structural analysis of the spatial organization of plasmonic NPs electrostatically adsorbed on thermoresponsive microgels and their evolution across the VPT. By combining electron microscopy, SAXS, and numerical simulations, supported by a simple toy model, we have unambiguously identified the many complex features occurring in the *S*(*q*) of the NPs, interpreting their modifications as a function of temperature. Our analysis reveals, besides the expected rescaling of NP–NP distances due to the microgel collapse, a clear tendency of the NPs to maximize their relative distance on the spherical surface of the microgel due to the underlying electrostatic repulsion. This results in increasingly ordered structures, as evidenced by spherical structure factor and Voronoi tessellation, albeit without reaching a crystalline arrangement. This result is particularly relevant, thanks to the paradigm shift introduced in interpreting microgels as dynamic substrates with precisely controllable curvature via external stimuli, which allows us to experimentally access, for the first time, the behavior of NPs confined on two-dimensional curved surfaces at the nanoscale. Moreover, the ability to modulate curvature and to monitor how particles rearrange consequently opens, in principle, the possibility of time-resolved experiments under dynamically evolving geometrical constraints. For instance, with appropriate modifications to the toy model able to account for ordered NP arrangements, this platform could enable, in the future, the experimental investigation of phase transitions on curved two-dimensional surfaces. Altogether, these aspects hold strong potential for advancing our understanding of curvature-mediated interactions and collective behavior in biologically relevant systems.

Importantly, we also provide a link between the structure of the microgel–NPs complexes and their optical properties, confirmed by electromagnetic full-wave simulations, establishing for the first time the relationship between the degree of plasmon coupling in the extinction spectra and the surface-to-surface distance between neighboring NPs. We plan to extend the present study to investigate the effects of NP size and their possible penetration into the polymer network down to the microgel core. This condition can be achieved, for instance, by using small NPs (*D* ≤ 5 nm) in combination with ionic microgels bearing charges distributed throughout the network. Both aspects are particularly relevant for applications as they offer additional degrees of freedom to finely tune and optimize the system’s optical properties.

In summary, our study provides a microscopic realization of particle assembly on reconfigurable curved surfaces at the nanoscale, with direct implications for plasmonics. At the same time, it sets the foundation for a broader exploration of curvature-mediated interactions and phase transitions in soft matter and biological systems and for the exploitation of the tunable optical properties of the NPs.

## 4 Methods

### 4.1 Microgel Synthesis

Our experiments are performed on cationic microgels with a fraction of crosslinker monomer *c* = 0.05 and of initiator *f*/2 = 0.01, synthesized by the surfactant-free radical polymerization previously detailed.^35,45^ We dissolve 1.25 g of NIPAM monomers (Sigma-Aldrich, MW = 113.16 Da) and the crosslinker *N*,*N*’-methylene-bis-acrylamide (BIS, Sigma-Aldrich, MW = 154.17 Da) in 148 mL of deionized water. Separately, the ionic initiator 2,2’-azobis­(2-methylpropionamidine) dihydrochloride (AIBA, Sigma-Aldrich, MW = 271.19 Da) is dissolved in 2 mL of water. The solution containing NIPAM and BIS is bubbled with argon for 30 min, and, after heating up to 70 ^◦^C, the initiator solution is added. At 70 ^◦^C, AIBA undergoes homolytic cleavage, forming two radicals, each including one amine group. Each radical reacts with a NIPAM monomer and produces a new radical, giving rise to the polymerization reaction. Therefore, after starting the reaction, amines of AIBA initiator remain attached to the backbone of the microgels, providing them positive charge due to protonation. After a 6-hour reaction, the obtained dispersion is cooled down to room temperature and filtered through glass wool. To prevent bacterial growth, NaN_3_ (Sigma-Aldrich, MW = 65.01 Da) is added at a concentration of 2 mM. The hydrodynamic radius of the microgels, measured by dynamic light scattering (DLS) at 25 ^◦^C, is *R*_*H*_ = 286 nm. The final number density of the microgels in the dispersion is evaluated to *n*_*mg*_ = 1.63 × 10^12^ mL^–1^ (volume fraction φ = 0.16) by viscosimetry measurements, as described in refs. 35 and 46.

### 4.2 Preparation of Microgel–Nanoparticles Samples

We use spherical gold NPs (Ted Pella) with a nominal diameter *D* = 20 nm and a number density *n*_*NP*_ = 7.0 × 10^11^ mL^–1^. A stabilizing citrate capping provides NPs with a negative charge, previously evaluated to *q*_*NP*_ = −35 *e*.^35^ The very low concentration guarantees that possible effects on the ionic strength of the final samples due to ion release from the NP surface are negligible. To prepare the samples, we separately dilute the microgel dispersion 250 times in 0.4 mM NaN_3_ and the NP solution in water to obtain the desired number ratio *n* = *n*_*NP*_/*n*_*mg*_. We then mix the two components and gently agitate the solution by hand. In the final samples, the number density of the microgels is *n*_*mg*_ = 3.26 × 10^9^ mL^–1^ and the concentration of NaN_3_ is 0.2 mM, which is low enough to exclude any effect of the ionic strength on the microgel swelling. Under the studied conditions, the Debye length is estimated to be ∼ 20 nm.

Since we are interested in studying plasmon coupling, which takes place for surface-to-surface distances between NPs of a few nanometers, we chose high *n* values to achieve sufficiently small distances and to avoid inducing aggregation of the microgel–NPs complexes. Preliminary DLS and extinction spectroscopy experiments show that these conditions are matched for *n* ≥ 100. Moreover, since at high *n,* only part of the NPs in the sample is actually incorporated within the microgel network due to electrostatic repulsion,^35^ we need to ensure that the portion of non-adsorbed NPs is not predominant to avoid compromising the outcomes of the experiments. A previous study of NP adsorption to microgels with *f* = 0.032 ^35^ shows that this requirement is well-fulfilled for *n* ≤ 300. Even though the microgels in this study are slightly less charged, we adhered to these limits, selecting *n* = 150 and *n* = 300. The good quality of the extinction spectra and SAXS curves acquired from the samples, which clearly show the features of NP adsorption and structuring onto the microgels, further supports this choice.

### 4.3 Extinction Spectroscopy

Extinction spectra in the UV–visible-NIR spectral range are acquired by including the samples in a 1 mm quartz cuvette and using a V-570 double-ray spectrophotometer (Jasco, Tokyo, Japan), equipped with a Peltier-thermostated holder EHC-505 (Jasco). The instrument has a spectral resolution of 0.1 nm in the UV–visible range and 0.5 nm in the NIR range. For measurements at varying temperatures, samples are allowed to thermalize for 5 min after each temperature change before acquisition.

To quantify the plasmon coupling from the acquired spectra, we identify the spectral region of coupled modes analogously to refs. 27 and 47 in the wavelength range from 570 to 800 nm (inset of Figure 1B), where only low-energy modes resulting from near-field interactions contribute to the extinction, and calculate their spectral weight. We thus define the coupling degree as

1 $$\Delta \frac{A_{C}}{A_{tot}}=\frac{A_{C}\left(T\right)}{A_{tot}\left(T\right)}-\frac{A_{C}^{\left(NP\right)}}{A_{tot}^{\left(NP\right)}}$$

where *A*_*tot*_(*T*) and *A*_*C*_(*T*) are the areas underlying the spectrum acquired at temperature *T* on the microgel–NPs samples, in the overall spectral range and in the region of coupled modes, respectively; $A_{tot}^{\left(NP\right)}$ and $A_{C}^{\left(NP\right)}$ are the corresponding areas computed from the reference spectrum of NP stock dispersion, which, due to its high dilution and colloidal stability, ensures the absence of plasmon coupling.

### 4.4 Electron Microscopy

For transmission electron microscopy, we use a Tecnai G^2^ 12 TWIN (FEI Company) setup that operates at 120 kV. The microscope is equipped with an electron energy loss filter (Biofilter, Gatan Inc.) and a slow-scan charge-coupled device camera (794 IF, Gatan Inc.). Before imaging, 20 μL of each sample is deposited on a 300-mesh copper grid covered by a thin amorphous carbon film. The deposition is done at a controlled temperature below (25^◦^C) or above (40^◦^C) the VPT. For better visualization, the samples deposited at 25^◦^C are stained with phosphotungstic acid by adding 10 μL of 2% aqueous solution (with pH adjusted to 7.3 using 1 N NaOH) to each deposition.

### 4.5 Small-Angle X-ray Scattering

For SAXS experiments, samples are filled in capillaries (1.5 mm diameter) and placed at a sample-to-detector distance of 3 m. The exposure time for acquisitions is set to 1 s, and 14 scattering patterns are acquired for each sample. Scattering patterns are recorded at 12 keV by using a two-dimensional EigerX 4M detector (Dectris, Baden, Switzerland). This allows measurements in the range of *q*-vector between 0.002 and 0.38 Å^–1^, where *q* is defined as *q* = (4π/λ)­sinθ, 2θ is the scattering angle, and λ is the wavelength of the radiation. Scattering patterns of an empty capillary and a capillary filled with water are recorded for normalization of the intensity to absolute units and background subtraction, respectively. Experiments are conducted at selected temperatures between 25 and 41 ^◦^C by employing a Huber Ministat 125 thermostat. After each temperature change, samples are left to thermalize for 5 min before measurements. The processing and averaging of the scattering patterns are performed using the software Foxtrot (SOLEIL software group and SWING beamline). When averaging, any scattering curve not perfectly superimposed with the overall set acquired, due to possible residual equilibration or other experimental perturbations, is discarded.

For a collection of particles, the scattered intensity *I*(*q*) can be expressed in terms of the form factor *P*(*q*) of single particles and the structure factor *S*(*q*) of the system as *I*(*q*) = *nv* ^2^Δρ^2^ *P*(*q*)*S*(*q*), where *n* and *v* are the number density and the volume of the scattering particles, and Δρ is the contrast in electron density ρ between particles and solvent. *S*(*q*) is the interference introduced by interparticle correlations and can be expressed in terms of the Fourier transform of the pair correlation function *g*(*r*) as

$$S\left(q\right)=1+\rho \int_{V}g\left(r\right)e^{-i\mathrm{q⃗}\cdot \mathrm{r⃗}}d\mathrm{r⃗}$$

Since for a dilute system of noninteracting scatterers, *S*(*q*) = 1, we directly measure the form factor of gold NPs in the stock solution.^29^ In microgel–NPs samples, given the extremely high contrast Δρ of gold compared to that of the polymer chains, it is reasonable to assume that the measured scattered intensities, with the chosen acquisition times, originate solely from the NPs present in the sample. We verify this assumption by measuring a microgel sample without NPs under the same experimental conditions, resulting in almost null scattered intensity. We can therefore simply derive the structure factor of NPs by dividing the scattered intensity measured for each sample by the form factor of NPs.

### 4.6 Dynamic Light Scattering

Distributions of hydrodynamic radius *R*_*H*_ are measured by DLS, employing a NanoZetaSizer apparatus (Malvern Instruments Ltd.) equipped with a He–Ne laser (5 mW power, 633 nm wavelength) that collects light in quasi-backscattering geometry at an angle of 173°. Decay times, extrapolated from the acquired intensity autocorrelation functions, are used to determine the distribution of diffusion coefficients, *D_T_,* of the particles. Diffusion coefficients are then converted to intensity-weighted distributions of *R*_*H*_ using the Stokes–Einstein relationship, *R*_*H*_ = *k*_*B*_ *T*/6πη*D*_*T*_, where *k*_*B*_ *T* is the thermal energy and η is the water viscosity. Temperature trends are measured using ascending ramps between 25°C and 41°C. After each temperature variation, the samples are kept thermalizing for 5 min before performing the measurement. Each value of *R*_*H*_ reported in this work is the average of a distribution obtained from at least 50 measurements. The associated error is the corresponding standard deviation.

### 4.7 Molecular Dynamics Simulations

We use coarse-grained microgels consisting of fully bonded, disordered polymer networks of *N* spherical beads with diameter σ and mass *m*, which set the length and mass units. NPs are also spherical beads of diameter *D* and mass *m*. Microgels of *N* = 112*k* or *N* = 14*k* monomers are prepared with the protocol previously reported in refs. 32 and 33, which was found to reproduce very well the experimental structure of the particles. After assembly, monomers interact via the bead–spring model, established by Grest and Kremer,^48^ which sums a steric repulsion term for all beads to a bond term for the connected ones. The first term is modeled by the Weeks–Chandler–Anderson (WCA) potential:

$$V_{\mathrm{WCA}}\left(r\right)=\left\{ \begin{array}{ll} 4\varepsilon \left[{\left(\frac{\sigma }{r}\right)}^{12}-{\left(\frac{\sigma }{r}\right)}^{6}\right]+\varepsilon & \mathrm{if}\;r\leq 2^{1/6}\sigma \\ 0 & \mathrm{if}\;r>2^{1/6}\sigma \end{array}\right.$$

where *r* is the center-to-center distance between a given pair of interacting particles, and ε sets the energy scale. The second term is the finitely extensible nonlinear elastic (FENE) potential:^34,49^

$$V_{\mathrm{FENE}}\left(r\right)=-\varepsilon k_{F}{R_{F}}^{2}\ln\left[1-{\left(\frac{r}{R_{F}\sigma }\right)}^{2}\right],r<R_{F}\sigma$$

with *R*_*F*_ = 1.5 and *k*_*F*_ = 15. Monomers are linked via the FENE potential to two neighbors, representing segments of NIPAM chains, whereas crosslinkers have a 4-fold valence. As for experiments, we use the molar fraction of crosslinker *c* = 0.05. Once assembled, bonds cannot break during the course of a simulation.

To model the VPT of microgels, we introduce an effective solvophobic potential *V*_α_, acting only between divalent monomers, which implicitly accounts for monomer–solvent interactions:^36^

$$V_{\alpha }\left(r\right)=\left\{ \begin{array}{lll} -\varepsilon \alpha & \mathrm{if} & r\leq 2^{1/6}\sigma \\ \frac{1}{2}\alpha \varepsilon \left[\cos\left(\gamma_{0}{\left(\frac{r}{\sigma }\right)}^{2}+\beta_{0}\right)-1\right] & \mathrm{if} & 2^{1/6}\sigma <r\leq R_{F}\sigma \\ 0 & \mathrm{if} & r>R_{F}\sigma \end{array}\right.$$ 2

where γ_0_ = π/(2.25–2^1/3^) and β_0_ = 2π – 2.25γ_0_. This is an attractive term modulated by the solvophobic parameter α, which plays the role of an effective temperature. Therefore, α = 0 represents good solvent conditions (at low temperature, below the transition), while as α rises, the attraction between monomers grows, leading to aggregation and microgel shrinkage; the overall behavior echoes the worsening of the monomer affinity to the solvent when temperature is increased.^32,50^

To mimic the ionic groups of AIBA monomers, we provide a fraction *f* of the microgel beads with a positive charge. Similar to experiments, we simulate microgels with a surface charge distribution, where charged beads are assigned randomly, but only in the exterior corona of the microgel, i.e., where the distance from the microgel center of mass is greater than *R*_*g*_. To ensure overall electroneutrality, for each charged monomer, we also insert an oppositely charged counterion, whose diameter is set to σ_*c*_ = 0.1σ.^34^ Counterions interact with each other and with microgel beads through the WCA potential. Additionally, all charged particles interact through the Coulomb potential:

$$V_{coul}\left(r\right)=\frac{q_{i}q_{j}\sigma }{e^{*2}r}\varepsilon$$

where *q*_*i*_ and *q*_*j*_ are the charges of the interacting beads (+e* for charged monomers of the microgel and –e* for counterions), being $e^{*}=\sqrt{4\pi \epsilon_{0}\epsilon_{r}\sigma \varepsilon }$ the reduced charge unit and ϵ_0_, ϵ_*r*_ the vacuum and relative dielectric constants, respectively. The particle–particle-particle-mesh method^51^ is adopted to appropriately account for the long-range nature of the Coulomb interactions. Following our previous works,^41,52^ charged monomers on the microgel do not interact with the solvophobic potential to ensure their maintained hydrophilic character in the whole investigated temperature range.

Finally, NPs are represented as single beads with a negative charge of *q* = −35e*. To maintain the same proportion between NPs and microgel sizes as in the experiments, we use two values for the NP diameter: *D* = 4σ for microgels with *N* = 112*k* and *D* = 2σ for *N* = 14*k*. Similarly to the process of assigning charge to AIBA monomers, an appropriate number of positive counterions (*q* = +e*, σ_*c*_ = 0.1σ) is added to preserve the overall neutrality of the system under all studied conditions. NPs, as all charged beads, interact with each other and with all other beads through the WCA and Coulomb potentials.

NVT simulations are performed using the LAMMPS package^53^ at a temperature fixed by *k*_*B*_ *T* = ε in a cubic box with side *L* and periodic boundary conditions. We choose *L* = 600σ for *N* = 112*k* and *L* = 300σ for *N* = 14*k*. The equations of motion are integrated with a time step Δ*t* = 0.002τ, where $\tau =\sqrt{m\sigma^{2}/\varepsilon }$ is the reduced time unit. We use the Nosé-Hoover thermostat in the constant NVT ensemble for equilibration (1000 τ) and the Velocity-Verlet algorithm in the constant-energy ensemble for the production runs (20000 τ). The latter is used to extract the equilibrium averages of the observables of interest.

The size of microgel–NPs complexes is characterized in terms of hydrodynamic radius *R*_*H*_, computed from simulations using the ZENO software.^54^ We include in the calculation all the monomers of the microgel and all NPs bound to charged monomers, identified as those with a distance from the closest charged monomer being lower than 1.25­(*D* + σ).

The structure factors of adsorbed NPs are calculated at each wavenumber *q* as

3 $$S\left(q\right)=\frac{1}{N_{\mathrm{ads}}}\overset{N_{\mathrm{ads}}}{\underset{i=1}{\sum }}\overset{N_{\mathrm{ads}}}{\underset{j=1}{\sum }}e^{-i\mathrm{q⃗}\cdot {\mathrm{r⃗}}_{ij}}$$

where *r⃗*_*ij*_ is the distance between the *i*-th and *j*-th NPs, and the sum is performed over all the NPs adsorbed to the mirogel, *N*_ads_, identified as those whose distance from the microgel center of mass is lower than *R*_*H*_ + *D*/2.

The spherical structure factors *S*( l ) are calculated using the geodesic distances between the adsorbed NPs, i.e., the angle determined by their positions on the unit spherical surface centered at the microgel’s center of mass. For an (*i*, *j*) pair of NPs, located at *r⃗*_*i*_ and *r⃗*_*j*_, the geodesic distance γ_*ij*_ is given by

$$\gamma_{ij}=2\arcsin\left(\frac{1}{2}|{\mathrm{r̂}}_{i}-{\mathrm{r̂}}_{j}|\right)$$

where *r̂*_*i*/*j*_ is the versor of *r⃗*_*i*/*j*_. *S*( l ) is then expressed as^6^

4 $$S\left(l\right)=1+\frac{2}{n}\underset{\left(i,j\right)}{\sum }P_{l}\left(\cos\gamma_{ij}\right)$$

where the sum is performed over all (*i*, *j*) pairs and *P*( l ) is the Legendre polynomial of degree l . Here, l plays an equivalent role to the wave vector *q* in ordinary space, with the difference that it can only take discrete values since the spherical surface that defines the geometry has finite size. To reduce statistical noise in the calculation of *S*( l ), we average results over five different microgel topologies.

The spherical Voronoi tessellation is computed, for each configuration, on the projection of the positions of adsorbed NPs on the unit sphere. The ordering of the Voronoi vertices around each position is obtained based on their convex hull.^55^ The distributions of the number *N*_*n*_ of nearest neighbors are the average over five different microgel topologies.

### 4.8 Toy Model

The toy model, sketched in Figure 4A, consists of a set of particles randomly positioned within a spherical shell. The particles mimic the NPs and the shell in which they are restrained represents the external corona of the microgel. The system is built from four parameters: the external radius *R* of the shell, its thickness *t*, the target number of particles to be placed within the shell, and the minimum surface-to-surface distance *d*_*min*_ between any two particles. The procedure to place the *j*th particle inside the shell is the following: first, (i) a random position within the shell is generated; next, (ii) the distance of this newly created position to all *j* – 1 previously inserted particles is calculated; and (iii) if the distance is larger than *D* + *d*_*min*_ for all present particles (here *D* = 18.6 nm is the NP diameter, obtained by the fit of Figure S4), the *j*th particle is assigned to this position. The procedure is repeated in the case of failing to allocate a new particle for a maximum number of 50*k* attempts for each particle. The limiting maximum number of attempts implies that in systems with high surface densities, the number *N*_*p*_ of particles actually placed in the shell may be lower than the target one.

For each set of the four parameters, we prepare 100 distinct configurations and compute the average of the corresponding structure factors according to eq 3. To account for the size polydispersity of experimental microgels, we consider a Gaussian distribution for the shell radius, defined by the mean value *R*_0_ and standard deviation σ_*R*_. We then extract from the distribution a different value of *R* for each of the 100 configurations; to keep the geometrical proportions constant, the shell thickness is also corrected by a factor *R*/*R*_0_. In this way, the features of the average structure factor result in more smoothed and therefore overlap better with experimental data. The individual effect of each parameter on the structure factor is analyzed in detail in Figures S8 and S9.

### 4.9 Full-Wave Electromagnetic Simulations

Simulated extinction spectra of gold NPs with diameter *D* = 18.6 nm randomly distributed on a spherical shell are obtained by numerically solving the complete set of Maxwell’s equations using the finite element method (FEM) implemented in the commercial software COMSOL Multiphysics^56^ with the frequency domain solver (radio frequency module). The geometry of the system for simulations is based on the configurations provided by the toy model described above. Specifically, a number *N*_*p*_ of particles are positioned at the coordinates given by the toy model, which ensure the best overlap of the structure factors with the experimental ones, measured at *T* = 25 °C and *T* = 41 °C. The geometric domain is enclosed by a perfectly matched layer (PML)^57^ with external and internal radii equal to $R_{\mathrm{PML}}^{\left(ext\right)}=2.5R$ and , respectively, where *R* is the external radius of the shell according to the toy model. The extinction cross-section is obtained by adding the absorption and scattering cross-sections. The former is determined by integrating the power loss density over the NP volume and the latter by integrating the Poynting vector over the spherical surface with the radius $R_{\mathrm{PML}}^{\left(int\right)}$ . Both contributions are normalized to the incident radiation. The incident field is a plane wave, linearly polarized along the *z* direction, expressed as $E_{\mathrm{IN}}=R\left(E_{0}e^{i\left(kx-\omega t\right)}\right)$ , where *E*_0_, ω, and *k* = 2π/λ are the amplitude, frequency, and wavevector, respectively, whereas *x* is the direction of the *k*-vector. The microgel is modeled as a nonuniform background with a radial dielectric permittivity, assuming a constant value in the region *r* < *R* and a Gaussian decay, as sketched in Figure S10. The numerical values of the refractive index at the two temperatures analyzed are calculated by considering the permittivity of the pNIPAM microgels^58^ appropriately scaled based on the water volume fraction present in the polymer network in the swollen and shrunken states, as determined in previous studies.^59,60^ For the optical functions of gold NPs and water, we used the data reported in the literature.^61,62^ All domains in the simulation box were meshed using tetrahedral elements, maintaining a maximum element size below λ/20 for the outer domain, where λ is the smallest wavelength used for the calculation of the extinction spectra, and below *D*/20 for the NPs.

## Supplementary Material



## Notes

Additional experimental analyses; additional numerical analyses; analysis of the toy model parameters; microgel model for electromagnetic simulations (PDF)

## References

[1] Thomson J. J. (1904). XXIV. On
the structure of the atom: an investigation of the stability and periods
of oscillation of a number of corpuscles arranged at equal intervals
around the circumference of a circle; with application of the results
to the theory of atomic structure. Lond. Edinb.
Dubl. Phil. Mag. J. Sci..

[2] Bowick M. J., Giomi L. (2009). Two-dimensional matter: order, curvature
and defects. Adv. Phys..

[3] Vitelli V., Lucks J. B., Nelson D. R. (2006). Crystallography
on curved surfaces. Proc. Natl. Acad. Sci. U.
S. A..

[4] Martín-Bravo M., Llorente J. M. G., Hernández-Rojas J., Wales D. J. (2021). Minimal
design principles for icosahedral virus capsids. ACS Nano.

[5] Fantoni R., Salari J. W., Klumperman B. (2012). Structure of colloidosomes with tunable
particle density: Simulation versus experiment. Phys. Rev. E.

[6] Božič A.
L., Čopar S. (2019). Spherical
structure factor and classification of hyperuniformity
on the sphere. Phys. Rev. E.

[7] Javidpour L., Božič A., Naji A., Podgornik R. (2021). Electrostatic
interactions between the SARS-CoV-2 virus and a charged electret fibre. Soft Matter.

[8] Meyra A. G., Zarragoicoechea G. J., Maltz A. L., Lomba E., Torquato S. (2019). Hyperuniformity
on spherical surfaces. Phys. Rev. E.

[9] Carenza L. N., Gonnella G., Marenduzzo D., Negro G., Orlandini E. (2022). Cholesteric
shells: two-dimensional blue fog and finite quasicrystals. Phys. Rev. Lett..

[10] Viveros-Méndez P., Méndez-Alcaraz J., González-Mozuelos P. (2008). Two-body correlations
among particles confined to a spherical surface: Packing effects. J. Chem. Phys..

[11] Bausch A., Bowick M. J., Cacciuto A., Dinsmore A., Hsu M., Nelson D., Nikolaides M., Travesset A., Weitz D. (2003). Grain boundary scars and spherical
crystallography. Science.

[12] Sun J. H., Zhang G. H., Plummer A., Martin C., Tanjeem N., Nelson D. R., Manoharan V. N. (2025). Colloidal
Crystallization on Cones. Phys. Rev. Lett..

[13] Singh N., Sood A., Ganapathy R. (2022). Observation
of two-step melting on
a sphere. Proc. Natl. Acad. Sci. U. S. A..

[14] Guerra R. E., Kelleher C. P., Hollingsworth A. D., Chaikin P. M. (2018). Freezing on a sphere. Nature.

[15] Larson R. G., Kotov N. A. (2015). Nonadditivity of
nanoparticle interactions. Science.

[16] Gao L., Dai X., Wu Y., Wang Y., Cheng L., Yan L.-T. (2024). Self-Assembly
at Curved Biointerfaces. ACS Nano.

[17] Fernandez-Nieves, A. ; Wyss, H. ; Mattsson, J. ; Weitz, D. A. Microgel Suspensions: fundamentals and Applications; John Wiley & Sons, 2011.

[18] Kelleher C. P., Guerra R. E., Hollingsworth A. D., Chaikin P. M. (2017). Phase behavior of
charged colloids at a fluid interface. Phys.
Rev. E.

[19] Gawlitza K., Turner S. T., Polzer F., Wellert S., Karg M., Mulvaney P., von Klitzing R. (2013). Interaction
of Gold Nanoparticles
with Thermoresponsive Microgels: Influence of the Cross-linker Density
on Optical Properties. Phys. Chem. Chem. Phys..

[20] Suzuki D., Nagase Y., Kureha T., Sato T. (2014). Internal structures
of thermosensitive hybrid microgels investigated by means of small-angle
X-ray scattering. J. Phys. Chem. B.

[21] Choe A., Yeom J., Shanker R., Kim M. P., Kang S., Ko H. (2018). Stretchable and wearable
colorimetric patches based on thermoresponsive
plasmonic microgels embedded in a hydrogel film. NPG Asia Mater..

[22] Sabadasch V., Wiehemeier L., Kottke T., Hellweg T. (2020). Core–shell
microgels
as thermoresponsive carriers for catalytic palladium nanoparticles. Soft Matter.

[23] Arif M., Farooqi Z. H., Irfan A., Begum R. (2021). Gold Nanoparticles
and Polymer Microgels: Last Five Years of their Happy and Successful
Marriage. J. Mol. Liq..

[24] Diehl F., Hageneder S., Fossati S., Auer S. K., Dostalek J., Jonas U. (2022). Plasmonic nanomaterials with responsive
polymer hydrogels for sensing
and actuation. Chem. Soc. Rev..

[25] Halas N. J., Lal S., Chang W.-S., Link S., Nordlander P. (2011). Plasmons in
strongly coupled metallic nanostructures. Chem.
Rev..

[26] Zygadlo K., Liu C.-H., Bernardo E. R., Ai H., Nieh M.-P., Hanson L. A. (2023). Correlating structural changes in
thermoresponsive
hydrogels to the optical response of embedded plasmonic nanoparticles. Nanoscale Adv..

[27] Aili D., Gryko P., Sepulveda B., Dick J. A., Kirby N., Heenan R., Baltzer L., Liedberg B., Ryan M. P., Stevens M. M. (2011). Polypeptide folding-mediated tuning of the optical
and structural properties of gold nanoparticle assemblies. Nano Lett..

[28] Li Z., Wang W., Yin Y. (2020). Colloidal assembly and active tuning
of coupled plasmonic nanospheres. Trends Chem..

[29] Capocefalo A., Bizien T., Sennato S., Ghofraniha N., Bordi F., Brasili F. (2022). Responsivity of Fractal
Nanoparticle
Assemblies to Multiple Stimuli: Structural Insights on the Modulation
of the Optical Properties. Nanomaterials.

[30] Liu H., Yang Z., Meng L., Sun Y., Wang J., Yang L., Liu J., Tian Z. (2014). Three-dimensional
and
time-ordered surface-enhanced Raman scattering hotspot matrix. J. Am. Chem. Soc..

[31] Caprara D., Ripanti F., Capocefalo A., Sarra A., Brasili F., Petrillo C., Fasolato C., Postorino P. (2020). DNA-functionalized
gold nanoparticle assemblies for Surface Enhanced Raman Scattering. Colloids Surf., A.

[32] Gnan N., Rovigatti L., Bergman M., Zaccarelli E. (2017). In Silico
Synthesis of Microgel Particles. Macromolecules.

[33] Ninarello A., Crassous J. J., Paloli D., Camerin F., Gnan N., Rovigatti L., Schurtenberger P., Zaccarelli E. (2019). Modeling Microgels
with a Controlled Structure Across the Volume Phase Transition. Macromolecules.

[34] Del
Monte G., Ninarello A., Camerin F., Rovigatti L., Gnan N., Zaccarelli E. (2019). Numerical Insights on Ionic Microgels:
Structure and Swelling Behaviour. Soft Matter.

[35] Brasili F., Del Monte G., Capocefalo A., Chauveau E., Buratti E., Casciardi S., Truzzolillo D., Sennato S., Zaccarelli E. (2023). Toward a unified
description of the electrostatic assembly of microgels and nanoparticles. ACS Appl. Mater. Interfaces.

[36] Soddemann T., Dünweg B., Kremer K. (2001). A generic computer model for amphiphilic
systems. Eur. Phys. J. E.

[37] Jain P. K., Huang W., El-Sayed M. A. (2007). On the
universal scaling behavior
of the distance decay of plasmon coupling in metal nanoparticle pairs:
a plasmon ruler equation. Nano Lett..

[38] Despert G., Oberdisse J. (2003). Formation
of micelle-decorated colloidal silica by
adsorption of nonionic surfactant. Langmuir.

[39] Oberdisse J. (2004). Small angle
neutron scattering and model predictions for micelle-decorated colloidal
silica beads. Phys. Chem. Chem. Phys..

[40] Lugo D., Oberdisse J., Karg M., Schweins R., Findenegg G. H. (2009). Surface
aggregate structure of nonionic surfactants on silica nanoparticles. Soft Matter.

[41] Del
Monte G., Truzzolillo D., Camerin F., Ninarello A., Chauveau E., Tavagnacco L., Gnan N., Rovigatti L., Sennato S., Zaccarelli E. (2021). Two-step deswelling in the Volume
Phase Transition of thermoresponsive microgels. Proc. Natl. Acad. Sci. U. S. A..

[42] Sönnichsen C., Reinhard B. M., Liphardt J., Alivisatos A. P. (2005). A molecular
ruler based on plasmon coupling of single gold and silver nanoparticles. Nature Biotechnol..

[43] Liu G., Wang D., Zhou F., Liu W. (2015). Electrostatic self-assembly
of Au nanoparticles onto thermosensitive magnetic core-shell microgels
for thermally tunable and magnetically recyclable catalysis. Small.

[44] Chang K., Yan Y., Zhang D., Xia Y., Chen X., Lei L., Shi S. (2023). Synergistic Bonding of Poly (N-isopropylacrylamide)-Based Hybrid
Microgels and Gold Nanoparticles Used for Temperature-Responsive Controllable
Catalysis of p-Nitrophenol Reduction. Langmuir.

[45] Truzzolillo D., Sennato S., Sarti S., Casciardi S., Bazzoni C., Bordi F. (2018). Overcharging and Reentrant
Condensation
of thermoresponsive Ionic Microgels. Soft Matter.

[46] Truzzolillo D., Roger V., Dupas C., Mora S., Cipelletti L. (2015). Bulk and Interfacial
Stresses in Suspensions of Soft and Hard Colloids. J. Phys.: condens. Matter.

[47] Chowdhury M. H., Julian A. M., Coates C. J., Coté G. L. (2004). Detection
of differences in oligonucleotide-influenced aggregation of colloidal
gold nanoparticles using absorption spectroscopy. J. Biomed. Opt..

[48] Grest G. S., Kremer K. (1986). Molecular Dynamics Simulation for Polymers in the Presence
of a Heat Bath. Phys. Rev. A.

[49] Kremer K., Grest G. S. (1990). Dynamics of Entangled Linear Polymer
Melts: A Molecular-dynamics
Simulation. J. Chem. Phys..

[50] Moreno A. J., Verso F. L. (2018). Computational investigation
of microgels: synthesis
and effect of the microstructure on the deswelling behavior. Soft Matter.

[51] Deserno M., Holm C. (1998). How to Mesh Up Ewald
Sums. I. A Theoretical and Numerical Comparison
of Various Particle Mesh Routines. J. Chem.
Phys..

[52] Del
Monte G., Camerin F., Ninarello A., Gnan N., Rovigatti L., Zaccarelli E. (2021). Charge affinity
and solvent effects in numerical simulations of ionic microgels. J. Phys.: Condens. Matter.

[53] Plimpton S. (1995). Fast Parallel
Algorithms for Short-range Molecular Dynamics. J. Comput. Phys..

[54] Juba D., Audus D. J., Mascagni M., Douglas J. F., Keyrouz W. (2017). ZENO: Software
for Calculating Hydrodynamic, Electrical, and Shape Properties of
Polymer and Particle Suspensions. J. Res. Natl.
Inst. Stand. Technol..

[55] Caroli, M. ; de Castro, P. M. ; Loriot, S. ; Rouiller, O. ; Teillaud, M. ; Wormser, C. Robust and efficient Delaunay triangulations of points on or close to a sphere. In Experimental Algorithms: 9th International Symposium, SEA 2010, Ischia Island, Naples, Italy Proceedings; Springer, 2010; Vol. 6049, pp. 462–473. 10.1007/978-3-642-13193-6_39.

[56] COMSOL. Multiphysics® v. 6.2; COMSOL AB: Stockholm, Sweden.

[57] Yushanov, S. ; Crompton, J. S. ; Koppenhoefer, K. C. Mie scattering of electromagnetic waves, Proceedings Of The COMSOL Conference, Cosmol Inc. Boston, 2013, 1–7

[58] Brasse Y., Müller M. B., Karg M., Kuttner C., König T. A., Fery A. (2018). Magnetic and electric resonances in particle-to-film-coupled functional
nanostructures. ACS Appl. Mater. Interfaces.

[59] Camerin F., Gnan N., Rovigatti L., Zaccarelli E. (2018). Modelling
realistic microgels in an explicit solvent. Sci. Rep..

[60] Bischofberger I., Trappe V. (2015). New aspects in the
phase behaviour of poly-N-isopropyl
acrylamide: systematic temperature dependent shrinking of PNiPAM assemblies
well beyond the LCST. Sci. Rep.

[61] Rakić A. D., Djurišić A. B., Elazar J. M., Majewski M. L. (1998). Optical
properties of metallic films for vertical-cavity optoelectronic devices. Appl. Opt..

[62] Laurens, J. E. ; Oughstun, K. E. Electromagnetic impulse response of triply-distilled water. In Ultra-Wideband Short-Pulse Electromagnetics 4 (IEEE Cat. No. 98EX112), 1998; pp. 243–264. 10.1109/UWBSP.1998.818959

