# Supplementary material for: Linking Structure and Optical Properties of Plasmonic Nanoparticles on Tunable Spherical Surfaces
Source: ACS Appl Mater Interfaces. 2025 Aug 11;17(33):47385–95. doi: 10.1021/acsami.5c11151 (PMC12371693; doi:10.1021/acsami.5c11151)
Supplement: Supplementary file 1 [file am5c11151_si_001.pdf]

Supporting Information: Linking structure and optical properties of plasmonic nanoparticles on tunable spherical surfaces

Francesco Brasili,[†,‡,#] Angela Capocefalo,[*,¶,#] Giovanni Del Monte,[†,§] Rodrigo Rivas-Barbosa,[‡] Javier Pérez,[‖] Edouard Chauveau,[⊥] Federico Bordi,[‡] Carlo Rizza,[¶] Domenico Truzzolillo,[⊥] Emanuela Zaccarelli,[*,†,‡] and Simona Sennato[*,†,‡]

†*Institute for Complex Systems, National Research Council, Piazzale Aldo Moro 5, 00185, Rome, Italy*

‡*Department of Physics, Sapienza University of Rome, Piazzale Aldo Moro 5, 00185, Rome, Italy*

¶*Department of Physical and Chemical Sciences, University of L'Aquila, Via Vetoio, Coppito, 67100, L'Aquila, Italy*

§*Soft Condensed Matter and Biophysics, Debye Institute for Nanomaterials Science, Utrecht University, Princetonplein 1, 3584 CC, Utrecht, The Netherlands*

‖*Synchrotron SOLEIL, L'Orme des Merisiers, Départementale 128, 91190 Saint-Aubin, France*

⊥*Laboratoire Charles Coulomb, UMR 5221, CNRS–Université de Montpellier, 34095, Montpellier, France*

#*F.B. and A.C. contributed equally to this work*

E-mail: angela.capocefalo@univaq.it; emanuela.zaccarelli@cnr.it; simona.sennato@cnr.it

S0.1 S1. Additional experimental analyses

Additional extinction spectra. We report in Fig. S1 the extinction spectra as a function of temperature between 25°C and 41°C of the NPs stock dispersion and of microgel–NPs complexes with number ratios $n = 15$ and $n = 300$. Due to the high dilution and colloidal stability of the stock sample, no plasmon coupling is observed for NPs alone (panel A) in the overall range of temperatures, as the extinction spectrum remains unaltered. For $n = 15$, the adsorbed NPs are few enough to remain far apart even above the VPT. Since in these conditions no plasmon coupling occurs (panel B), this sample is a reference to analyze the effects on the LSPR caused by water expulsion from the polymer network and consequent chain densification across the VPT. Hence, the observed shift of the LSPR, up to 8 nm, can be attributed solely to the variation of refractive index at the interface of the NPs with increasing temperature. At larger n values, the maximum shift is higher, reaching 10 nm for $n = 300$ (panel C) and 16 nm for $n = 150$ (Fig. 1A), pointing to a contribution from plasmon coupling.

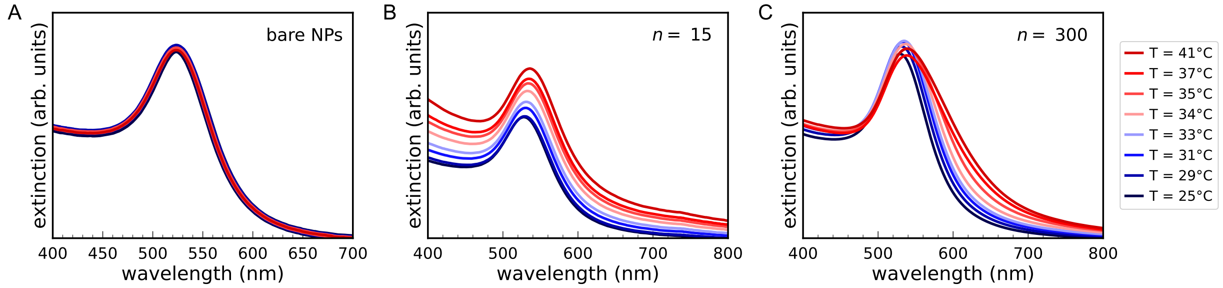

Figure S1: Extinction spectra of the NPs stock dispersion (A) and of the microgel–NPs complexes as a function of temperature for $n = 15$ (B) and $n = 300$ (C).

Experimental swelling curve of the microgels The experimental swelling curve of the microgels, obtained plotting R_H as a function of temperature is reported in Fig. S2. Based on it, we extrapolated the transition temperature $T_C = 33.3 \pm 0.3$ °C, by fitting data to the sigmoidal-like function:

$$y = y_\infty + \frac{y_0 - y_\infty}{1 + e^{\frac{x - T_C}{\Delta T}}} \quad . \tag{S1}$$

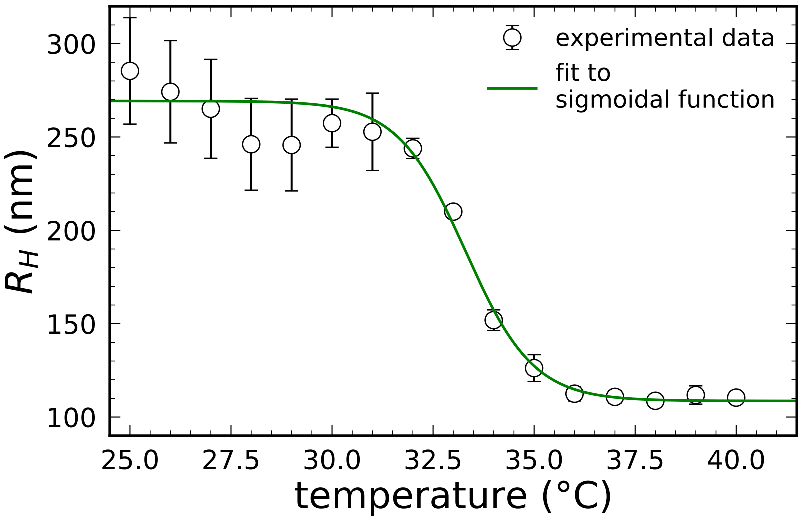

Figure S2: Swelling curve of the microgel and best fit to a sigmoidal-like function.

Additional electron microscopy images. We analyzed the arrangement of NPs in microgel–NPs samples by transmission electron microscopy. Representative images for the samples with $n = 150$ and $n = 300$ are shown in Fig. S3. This analysis reveals that, in both samples, NPs tend to localize in the peripheral regions of the microgel corona. Moreover, the NPs adsorb to the different microgels with low variability throughout the sample.

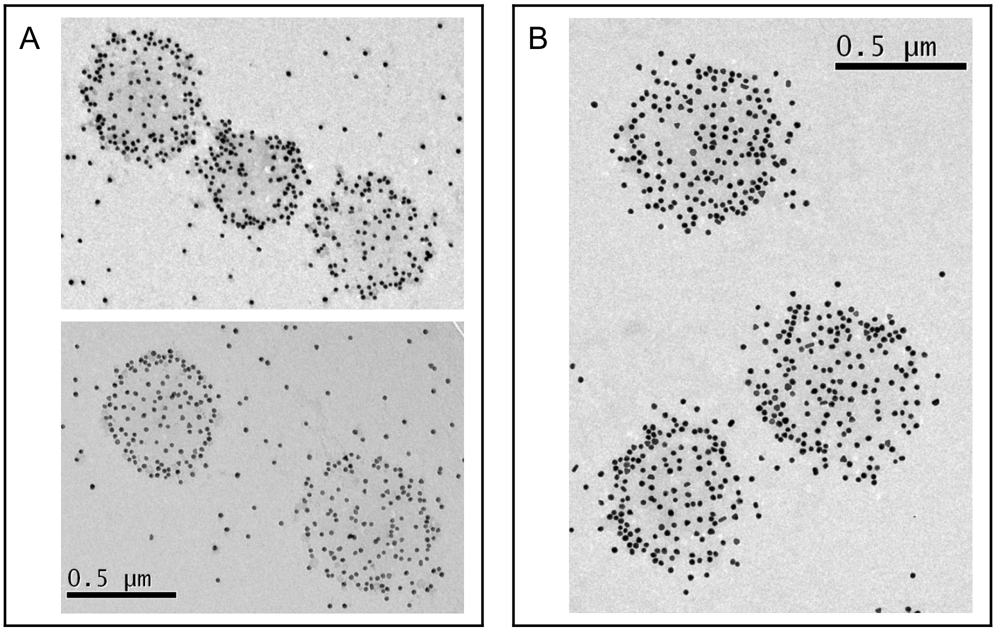

Figure S3: Electron microscopy images of the microgel–NPs complexes for $n = 150$ (A) and $n = 300$ (B).

NP form factor and structure factors of samples with $n = 300$ The form factor of gold NPs is directly measured by SAXS on dilute samples, with number density $n_{NP} = 7.0 \times 10^{11}$ mL^{-1}. The acquired scattering curve is reported in Fig. S4A. We fitted the curve to a spherical form factor model[1] with log-normal particle size distribution, to evaluate the radius of NPs to 9.3 nm with 10% polydispersity. The good overlap between the two curves also ensures the validity of the assumption that there is no interaction between the dispersed NPs, thus the scattering curve represents their form factor. In Fig. S4B, we report the structure factors of NPs measured on microgel–NPs samples with number ratio $n = 300$ as a function of temperature between 25 °C and 41 °C.

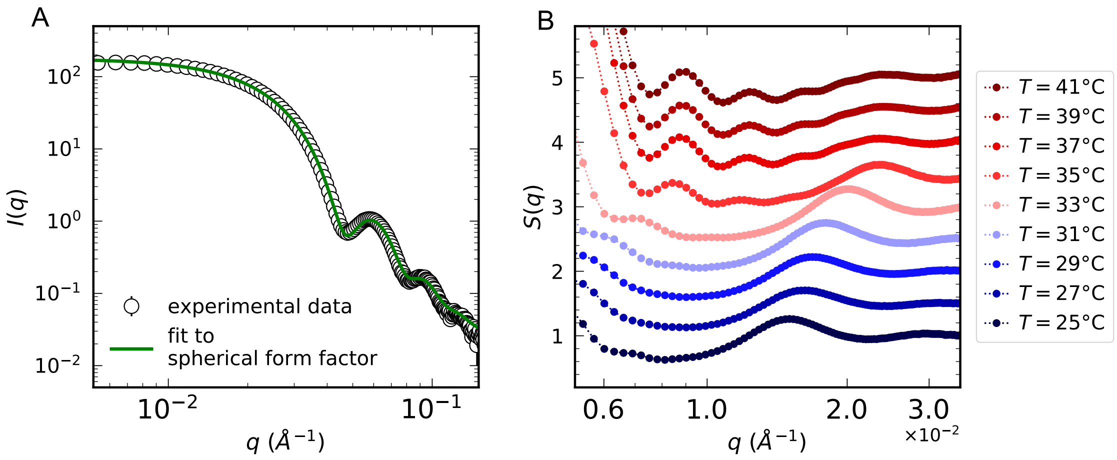

Figure S4: (A) Experimental form factor of gold NPs at $T = 25$ °C and best fit to a sphere model. (B) Structure factors of NPs as a function of temperature for the microgel–NPs sample with $n = 300$.

S0.2 S2. Additional numerical analyses

Numerical swelling curve of of the microgel. The swelling curve of the simulated microgel for $N = 112k$ and $f = 0.02$, as obtained by plotting R_H as a function of α is reported in Fig. S5A. Based on it, we extrapolate the equivalent transition temperature $\alpha = 0.64 \pm 0.02$, fitting the data to the sigmoidal-like function of Eq. S1.

Full-range structure factors for $n = 150$. The full q-range numerical structure factors calculated from the simulations with $N = 112k$ and $n = 150$ are shown in Fig. S5B, for $\alpha = 0$ and $\alpha = 2$. In particular, the features of the overall microgel–NPs system emerge at low-q, where curves are characterized by high oscillations. Proceeding from the electron microscopy analyses, we assumed that NPs are incorporated only in the microgel corona, therefore we employed a spherical shell model form factor to fit the curves. The model is directly derived from the difference between the amplitudes of two spherical form factors with same contrast and radii R_{out} and R_{in}, respectively:

$$P_{\text{shell}}(q) = A * \left[V_{tot} \frac{\sin(qR_{out}) - qR_{out}\cos(qR_{out})}{(qR_{out})^3} - V_{core} \frac{\sin(qR_{in}) - qR_{in}\cos(qR_{in})}{(qR_{in})^3} \right]^2 , \tag{S2}$$

where $V_{tot} = \frac{4}{3}\pi R_{out}^3$, $V_{in} = \frac{4}{3}\pi R_{in}^3$ and A is factor depending on the volume fraction and contrast of the shell. We fitted the numerical structure factors to Eq. S2, limiting to the low-q region. The resulting curves are superimposed on the data in the figure (continuous line), showing that this simple model successfully captures all the features of the NP structure factors at this length scale. The obtained values of the model radii are $R_{in} = (37.5 \pm 0.3)\,\sigma$ and $R_{out} = (48.7 \pm 0.3)\,\sigma$, for $\alpha = 0$, and $R_{in} = (28.3 \pm 0.2)\,\sigma$ and $R_{out} = (34.7 \pm 0.1)\,\sigma$, for $\alpha = 2$. However, the extension of the model to higher q-values (dotted line) clearly deviates from the numerical data, indicating the need for more detailed analyses of the NP structure and organization at the scale of interparticle interactions.

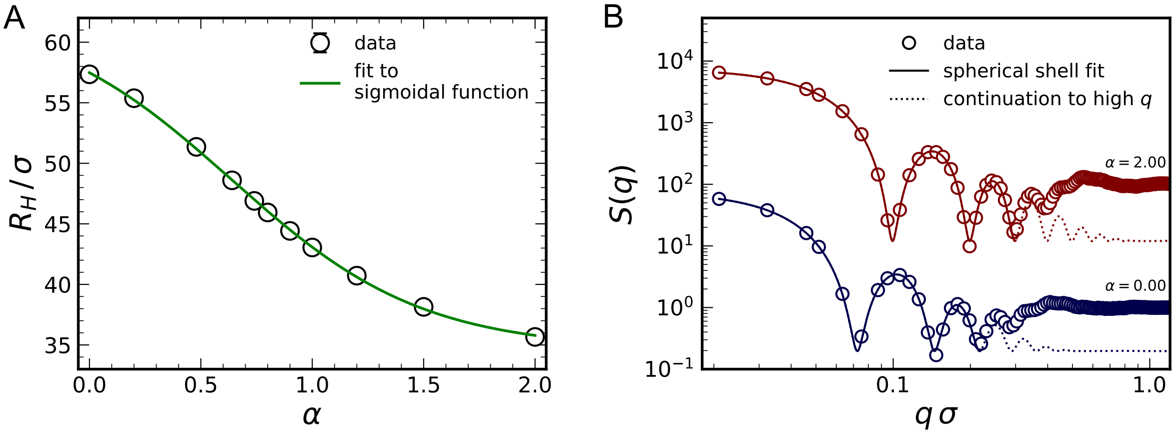

Figure S5: (A) Swelling curve, R_H as a function of α, of the microgel with $N = 112k$ and $f = 0.02$, and best fit to a sigmoidal-like function. (B) Full q-range numerical structure factors for $N = 112k$ and $n = 150$ (circles), with corresponding fits to the spherical shell form factor of Eq. S2 in the low-q region (continuopus line) and continuation of the curve in the overall q-range (dotted line), for $\alpha = 0$ (blue) and $\alpha = 2$ (red).

Additional numerical structure factors. The NPs structure factors for the microgels with $N = 112k$ and $f = 0.02$ at different NP/microgel number ratios and for different microgel compositions ($N = 14k$ and $f = 0.02$, $N = 14k$ and $f = 0.16$, $N = 112k$ and $f = 0.01$) at fixed $n = 150$ are reported as a function of α in Fig. S6. In all the cases considered, the high oscillations at low q are followed by smaller peaks at intermediate q (highlighted in light blue). These peaks are present for all the effective temperature, in contrast to experiments.

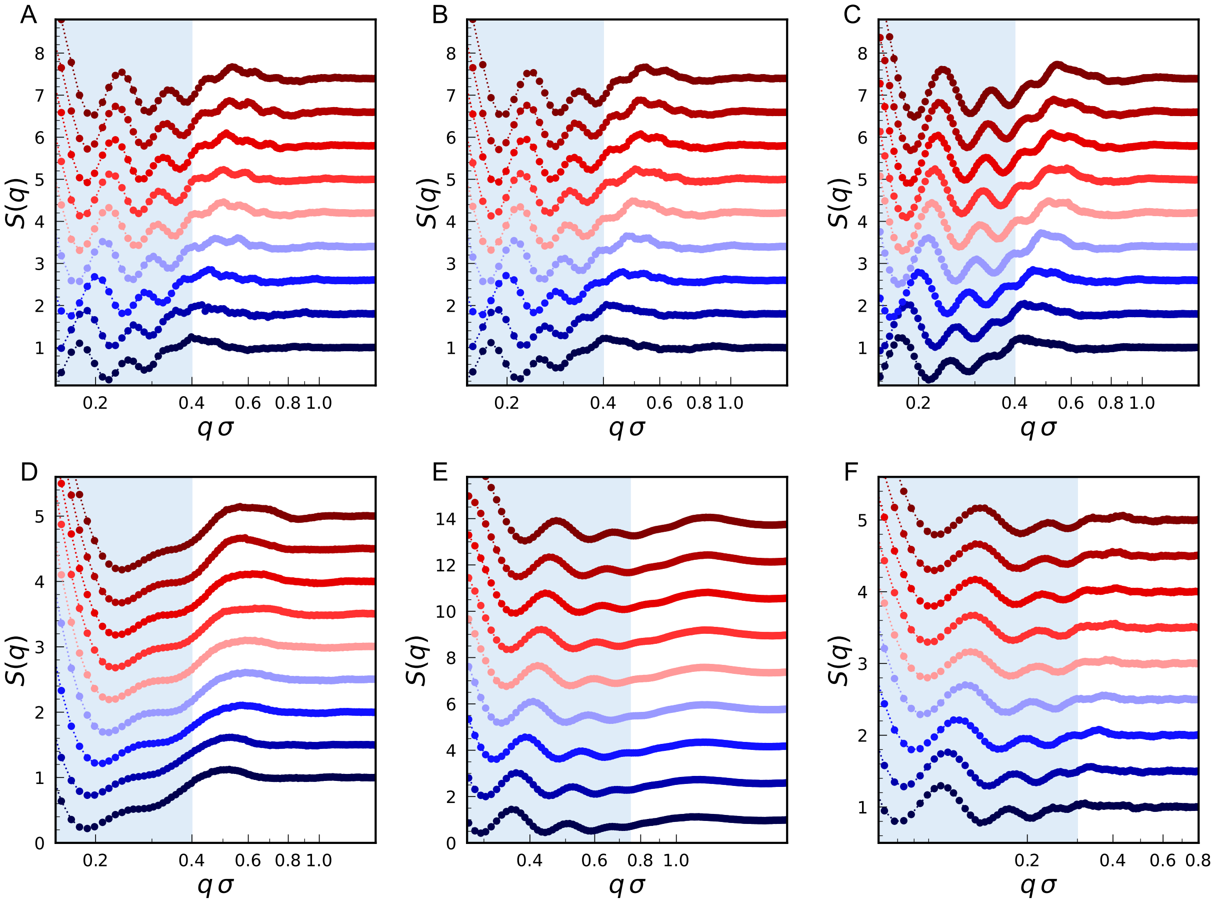

Figure S6: Structure factors at varying α for different microgel–NPs systems: microgel with $N = 112k$ and $f = 0.02$, for $n = 80$ (A), $n = 100$ (B) and $n = 300$ (C), and microgels with $N = 14k$ and $f = 0.02$ (D), $N = 14k$ and $f = 0.16$ (E), $N = 112k$ and $f = 0.01$ (F) at fixed $n = 150$. The color-coding is the same as in Fig. 2.

Additional spherical structure factors. The spherical structure factors of the adsorbed NPs for the system with $N = 112k$, $f = 0.02$ and $n = 150$, computed according to Eq. 9, are reported as a function of α in Fig. S7A. We find that $S(l)$ is characterized by a main peak for ~ 16, followed by smooth oscillations. Additionally, it approaches a low value as $l \to 0$. The T-dependence of $S(l)$ shows that the main peak becomes sharper, shifting to lower l as T increases. The same variations are more pronounced in the case of the microgel with $N = 112k$ and $f = 0.16$ reported in Fig. 3C. To sum up, the shift to low wavevectors of all structure factors clearly indicate that the NPs increase their relative (geodesic) distance as the microgels undergo the VPT.

In order to better assess the role of electrostatic interactions, we performed simulations on the same microgel interacting with NPs with different charges. We considered the two additional values of the NP charge, $q = -5\,e^*$ and $q = -10\,e^*$. The plots of $S(l)$ for the three values of q are reported in Figs. S6B and C, for $\alpha = 0.00$ and $\alpha = 2.00$, respectively. Noteworthy, the main peak of the curve, that becomes narrower and more pronounced as α increases above the VPT, is observable only for $q = -35\,e^*$, when the number of adsorbed NPs is higher and the electrostatic repulsion between them is stronger. This highlights the important role of electrostatic interactions in determining the spatial arrangement of the adsorbed NPs and its modification across the VPT.

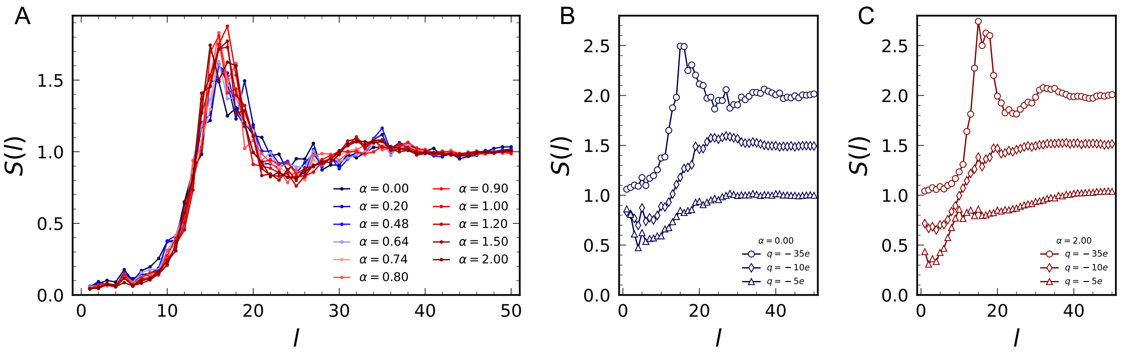

Figure S7: Spherical structure factors at varying α for the microgel–NPs complex with $N = 112k$, $f = 0.02$ and $n = 150$ (A). Spherical structure factors for the same microgel at varying the NP charge q, below (B, $\alpha = 0.00$) and above (C, $\alpha = 2.00$) the VPT of the microgel.

S0.3 S3. Analysis of the toy model parameters

To achieve a full comprehension of the structure factors of the NPs adsorbed to microgel, we varied each of the four parameters of the toy model independently and we analyzed how it affects the features of the $S(q)$. The trends at varying the shell parameters R_0, t and σ_R are shown in Fig. S8, those at varying the NPs parameters N_p and d_{min} are shown in Fig. S9.

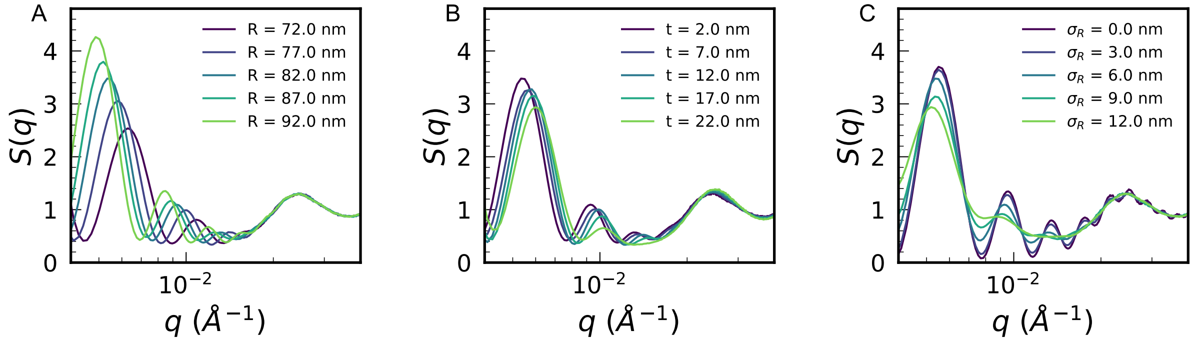

Figure S8: Structure factors of the toy model, consisting in particles with random positions within a spherical shell, at varying independently each geometrical parameter of the shell R (A), t (B) and σ_R (C). The following values are used for the parameters that are kept constant: $R = 82$ nm, $t = 2$ nm, $\sigma = 6$ nm, $N_p = 150$ and $d_{min} = 8$ nm.

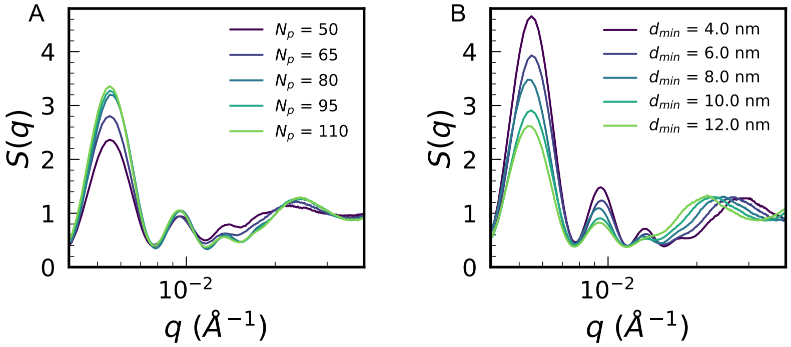

Figure S9: Structure factors of the toy model, consisting in particles with random positions within a spherical shell, at varying independently each parameter of the NPs, N_p (A) and d_{min} (B). The following values are used for the parameters that are kept constant: $R = 82$ nm, $t = 2$ nm, $\sigma = 6$ nm, $N_p = 150$ and $d_{min} = 8$ nm.

S0.4 S4. Microgel model for electromagnetic simulations

To perform full-wave electromagnetic simulations, we modelled the microgel as a non-uniform background with a radial dielectric permittivity, assuming a constant value for the microgel core and a Gaussian decay for its corona. The core is the region $r < R$, where R is the external radius of the shell according to the toy model. The numerical values of the refractive index at the two temperatures analyzed are calculated by scaling the permittivity of the pNIPAM microgels[2] to the corresponding fractions of water contained in the polymer network.[3,4] The radial profile of the microgel refractive index is sketched in Fig. S10.

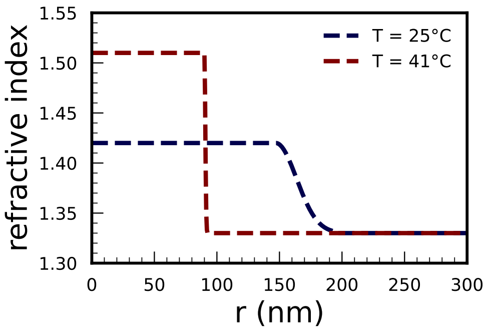

Figure S10: Radial profiles of the microgel refractive index used in full-wave simulations.

References

(1) Guinier, A.; Fournet, G. Small-angle scattering of X-rays. 1955.

(2) Brasse, Y.; Müller, M. B.; Karg, M.; Kuttner, C.; König, T. A.; Fery, A. Magnetic and electric resonances in particle-to-film-coupled functional nanostructures. *ACS applied materials & interfaces* **2018**, *10*, 3133–3141.

(3) Camerin, F.; Gnan, N.; Rovigatti, L.; Zaccarelli, E. Modelling realistic microgels in an explicit solvent. *Scientific reports* **2018**, *8*, 14426.

(4) Bischofberger, I.; Trappe, V. New aspects in the phase behaviour of poly-N-isopropyl acrylamide: systematic temperature dependent shrinking of PNiPAM assemblies well beyond the LCST. *Scientific reports* **2015**, *5*, 15520.
